# MicroRNA-125b exerts antitumor functions in cutaneous squamous cell carcinoma by targeting the STAT3 pathway

**DOI:** 10.1186/s11658-020-00207-y

**Published:** 2020-03-05

**Authors:** Ke Tian, Wanggen Liu, Jing Zhang, Xiaoyi Fan, Jingyuan Liu, Nan Zhao, Chunxia Yao, Guoying Miao

**Affiliations:** 1 Department of Dermatology, Affiliated Hospital of Hebei University of Engineering, Handan, 056002 China; 2 Department of Histology and Embryology, Preclinical Medicine College, Xinxiang Medical University, Xinxiang, 453003 China; 3 Department of Pathology, Medical School, Hebei University of Engineering, Handan, 056002 China

**Keywords:** microRNA-125b, Signal transducer and activator of transcription (STAT) 3, Cutaneous squamous cell carcinoma

## Abstract

**Background:**

MicroRNA-125b (miR-125b) is downregulated in human cutaneous squamous cell carcinoma (CSCC). However, its function in CSCC has yet to be extensively explored. Here, we analyze the relationship between signal transducer and activator of transcription 3 (STAT3) and miR-125b in CSCC.

**Methods:**

Western blotting and quantitative RT-PCR were used to determine the expression of the miR-125b–STAT3 axis in human CSCC tissues and cell lines. The direct regulatory effect of miR-125b on STAT3 expression was assessed using a luciferase reporter gene assay and RNA immunoprecipitation assay. The MTT assay and flow cytometry were used to determine the role of the miR-125b–STAT3 axis in CSCC cell proliferation and apoptosis.

**Results:**

MiR-125b expression levels were significantly lower in CSCC cell lines and tissues than in normal cell lines and tissues. STAT3 was identified as the direct target of miR-125b. Upregulation of miR-125b and downregulation of STAT3 suppressed cell proliferation and promoted cell apoptosis. Cyclin D1 and Bcl2 were identified as the downstream targets of the miR-125–STAT3 axis.

**Conclusions:**

Our findings indicate that miR-125b acts as a tumor suppressor in CSCC by targeting the STAT3 pathway. This observation increases our understanding of the molecular mechanisms of CSCC. Therapies aimed at activating miR-125b or inhibiting STAT3 signaling should be explored as potential treatments for CSCC.

## Background

Cutaneous squamous cell carcinoma (CSCC), which derives from the keratinocytes, is the second most common type of human non-melanoma skin cancer in the world [1]. Classical risk factors for the occurrence of CSCC include age, race, ultraviolet radiation exposure, skin phototype and immunosuppression [2]. It has a demonstrated epidemiological rise in recent decades [3]. Although CSCC usually displays benign clinical behavior and can be cured by surgical excision, about 8% of patients with CSCC develop a recurrence and 5% patients present metastasis within 5 years. The prognosis for metastatic CSCC is poor, and its one-year disease-specific survival is 44–56% [4]. A deeper understanding of the molecular mechanisms underlying the biological behavior of CSCC will provide important clues to improve the CSCC diagnosis and treatment.

MicroRNAs (miRNAs) are a family of ~ 23 nt long endogenous non-coding small RNAs. They control gene expression by binding with the 3′-untranslated regions (UTRs) of target mRNAs and then blocking translation or degrading mRNAs [5, 6]. They are implicated in a variety of physiological and pathological processes, including development, differentiation, proliferation, apoptosis and immune responses [7–9].

It has been shown that miRNAs are involved in the genesis and development of tumors [10]. They can promote carcinogenesis or prevent cancer development, depending on the roles of their target genes. Generally, oncogenic miRNAs are upregulated in cancers, while tumor suppressor miRNAs are downregulated [11].

MicroRNA-125b (miR-125b) has many known target genes in tumors, including Bcl2, MMP13, CDK6, c-JUN, IGFR1 and ERBB2/3 [12]. It is downregulated in CSCC relative to its expression in healthy skin [13]. Its overexpression in CSCC cell lines can inhibit CSCC cell proliferation and invasion through targeting MMP13 [13]. Our knowledge of the underlying mechanism of miR-125b in the formation and progression of CSCC remains inadequate.

Signal transducer and activator of transcription 3 (STAT3) plays important roles in the genesis and development of many types of cancer [14]. For instance, STAT3 plays a crucial role in the initiation and progression of epithelial carcinogenesis [15]. Its activation increases migration and invasion of bladder cancer cells [16]. More importantly for this study, miR-125b has been found to suppress osteosarcoma cell proliferation and migration by downregulating STAT3 [17]. However, whether miR-125b regulates STAT3 in CSCC tumorigenesis has yet to be clarified.

Here, we validate STAT3 as the direct target gene of miR-125b in human CSCC cells. We also show that miR-125b overexpression and STAT3 knockdown can suppress CSCC cell proliferation and induce cell apoptosis. Cyclin D1 and Bcl2 are established as the downstream targets of the miR-125b–STAT3 axis. This study is helpful to understand the carcinogenesis of CSCC and may give rise to a novel diagnosis and treatment strategy.

## Methods

### Cell culture and transfection

A human normal skin cell line (HaCaT) and three kinds of CSCC cell lines (A431, SCC13 and SCL-1) were ordered from the Cell Bank of the Chinese Academy of Sciences. Cells were cultured in RPMI-1640 medium with 10% fetal bovine serum (FBS; Gibco), 100 U/ml penicillin and 0.1 g/ml streptomycin (Sigma) in a humidified atmosphere with 5% CO_2_ at 37 °C.

Cells were seeded onto 6-well plates at a density of 3 × 10^5^ cells/per well and cultured overnight. When cell growth reached 80% confluence, the indicated plasmids, miR-125b mimics or scramble control miRNAs (GenePharma) were transiently transfected into the cells using Lipofectamine 2000 (Thermo Fisher Scientific) according to the manufacturer’s instructions. After incubation at 5% CO_2_ and37°C for 48 h, the transfected cells were used for further experiments.

### CSCC tissue samples

A total of 32 pairs of CSCC samples and corresponding adjacent non-tumor samples were obtained from CSCC patients at the Affiliated Hospital of Hebei University of Engineering between July 2017 and December 2018. Written informed consent was obtained from all patients before the experiments. All samples were snap-frozen in liquid nitrogen immediately after surgery and stored at − 80 °C until needed. All sample natures (CSCC or healthy) were confirmed via histopathological analysis. The study was approved by the Ethics Committee of Hebei University of Engineering (Approval Number: HUE-M-2017-026, Date: 2017.7.9; Handan, China) and performed following the principles of the Declaration of Helsinki.

### Western blotting

Total proteins were extracted from each tissue and cell line using RIPA lysis buffer and then quantified with a BCA kit (Pierce). A total of 40 μg proteins were loaded into each lane, separated using 10% SDS-PAGE and then transferred to PVDF membranes (Millipore). Membranes were blocked with 5% nonfat milk in TBST for 1 h at room temperature and then incubated with specific primary antibodies (Santa Cruz) overnight at 4 °C. After three 5-min washes with TBST, the membranes were incubated with secondary antibodies conjugated with HRP (Santa Cruz) for 1 h at room temperature. Finally, protein blots were visualized via ECL (Pierce). GAPDH was used as a loading control.

### RNA extraction and quantitative RT-PCR

Total mRNAs were extracted from tissues and cells using an RNAiso Plus Kit (Takara) and total miRNAs using an miRNeasy Mini Kit (Qiagen). cDNA was synthesized using PrimeScript RT Master Mix (Takara). Real-time PCR was performed on an ABI 7900 Fast PCR system (Applied Biosystems) using SYBR Green Supermix (Qiagen). U6 and GAPDH were respectively used to normalize miRNA and mRNA expressions. The 2^–ΔΔCt^ method was used to calculate the relative mRNA or miRNA expression levels. All experiments were plated in triplicate and performed three times. The primer sequences [17] were:

miR-125b forward, 5′-TCCCTGAGACCCTAACTTGTGA-3′;

miR-125b reverse, 5′-AGTCTCAGGGTCCGAGGTATTC-3′;

U6 forward, 5′-CTCGCTTCGGCAGCACA-3′;

U6 reverse, 5′-AACGCTTCACGAATTTGCGT-3′;

STAT3 forward, 5′-CTGTGTGACACCAACGACCT-3′;

STAT3 reverse, 5′-CACTCCGAGGTCAACTCCAT-3′;

GAPDH forward, 5′-GAAGGTGAAGGTCGGAGTC-3′;

GAPDH reverse, 5′-GAAGATGGTGATGGGATTTC-3′.

### Luciferase report gene assay

The predicted 3′-UTR sequences of STAT3 encompassing the miR-125b binding sites were amplified via PCR and subcloned into a dual-luciferase reporter construct pMIR-GLO (Promega) to form the pMIR-GLO-STAT3-WT reporter vector. The mutant reporter vector pMIR-GLO-STAT3-MUT was obtained using a site-directed mutagenesis kit (New England Biolabs). Cells of a CSCC cell line (SCC13) were co-transfected with miR-125b mimics or scramble control and with pMIR-GLO-STAT3-WT or MUT reporter vector using Lipofectamine 2000 (Thermo Fisher Scientific). After culture for 48 h, luciferase activities were analyzed using the Dual-Luciferase Reporter Assay System (Promega). All experiments were plated in triplicate and performed three times.

### Cell proliferation assay

After 48 h transient transfection, cells were harvested and seeded at 3000 cells/well into 96-well plates. An MTT assay was used to analyze cell proliferation at 24, 48, 72 and 96 h. Briefly, 20 μl of MTT solution (5 mg/ml) was added into each well and incubated at 37 °C until purple precipitates were visible. Then, MTT medium was removed, and 100 μl of DMSO was added to dissolve the crystals. The absorbance was read at 490 nm with a microplate reader (Bio-Rad). All experiments were plated in triplicate and performed three times.

### Cell cycle and apoptosis analysis by flow cytometry

Three kinds of CSCC cells were seeded at 3 × 10^5^ cells/well into 6-well plates the day before transfection. After 48 h transfection, the cells were collected and rinsed three times with ice-cold PBS. Then, the cells were stained with propidium iodide. Cell cycle and apoptosis data were measured using a BD FACScan Flow Cytometer equipped with Cell-Quest software (Becton Dickinson).

### RNA immunoprecipitation assay

Lysed cell extracts were immunoprecipitated with anti-human Argonaute-2 (Ago2) antibody or mouse IgG using protein G Sepharose Beads. After elution from the beads, the immunoprecipitated RNA was extracted and measured via quantitative RT-PCR to verify the enrichment of binding sites of STAT3.

### Statistical analysis

The data are reported as means ± standard deviation (SD). Student’s t-test was used for statistical comparisons of two groups, and one-way or two-way analysis of variance (ANOVA) was used for statistical comparisons of multiple groups. SPSS software version 17.0 was used in all cases. *p* < 0.05 was considered a statistically significant difference.

## Results

### MiR-125b and STAT3 expression in CSCC tissues and cell lines

**Fig. 1:**
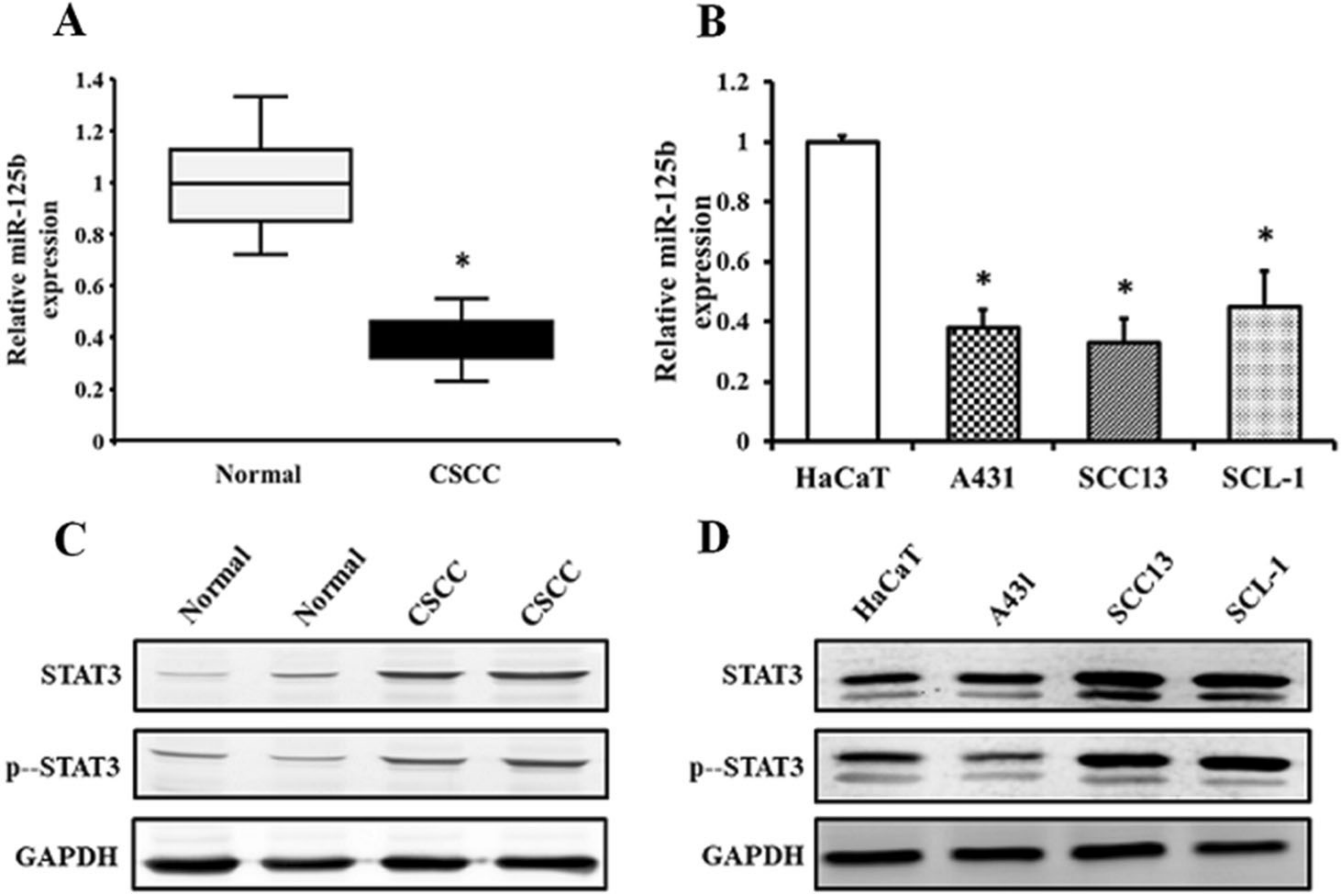
MiR-125b and STAT3 expression in CSCC tissues and cell lines. **a** MiR-125b expression in CSCC tissues and adjacent normal tissues. **b** MiR-125b expression in cells of CSCC cell lines (A431, SCC13 and SCL-1) and a normal skin cell line (HaCaT). **c** The STAT3 protein level in CSCC tissues and adjacent normal tissues. **d** The STAT3 total and phosphorylated protein levels in cells of CSCC cell lines (A431, SCC13 and SCL-1) and a normal skin cell line (HaCaT). *p < 0.05 in comparison with normal tissues or cell lines

To discover the functional role of miR-125b in CSCC, we first determined its expression levels in CSCC tissues and adjacent normal tissues, as well as in cells of three CSCC cell lines (A431, SCC13 and SCL-1) and the normal human cell lin HaCaT. The miR-125b expression levels were significantly lower in the CSCC tissues and cells than in the healthy skin tissues and cells (Fig. 1a and b), indicating that miR-125b may have a tumor suppressor effect.

We also determined the expression levels of STAT3 in CSCC tissues and cell lines. The mRNA levels of STAT3 tested via quantitative PCR did not have any significant difference to those in normal skin tissues and cell lines (data not shown). However, the total protein levels of STAT3 and the levels of phosphorylated STAT3 (p-STAT3) increased significantly in CSCC tissues and cells (A431, SCC13 and SCL-1) relative to those in normal tissues and HaCaT cells (Fig. 1c and d). The inverse expression pattern between miR-125b and STAT3 suggests that STAT3 might be the potential target of miR-125b.

### MiR-125b directly targets STAT3 in CSCC cells

**Fig. 2:**
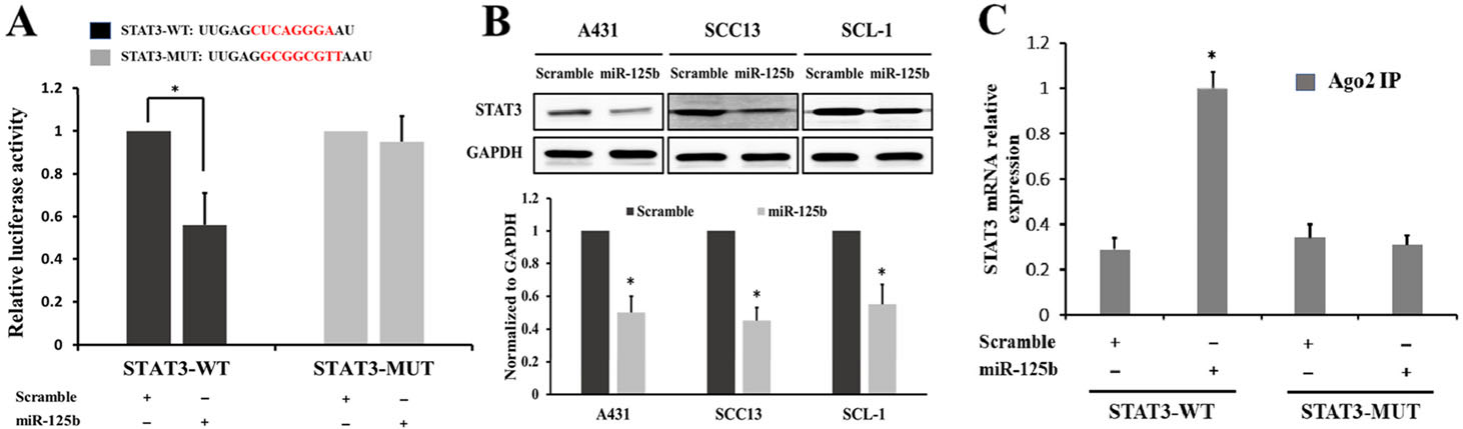
STAT3 is a direct target of miR-125b. **a** The binding sequence for miR-125b in the 3′-UTR of STAT3 and the mutations are indicated in red. The binding relationship between miR-125b and STAT3 in SCC-13 cells (an CSCC cell line) was analyzed using the dual-luciferase system. **b** Western blotting was used to determine STAT3 protein expression in A431, SCC13 and SCL1 cells transfected with the miR-125b mimic or scrambled control. **c** Immunoprecipitation–quantitative PCR was used to evaluate the direct interaction between miR-125b and STAT3. *p < 0.05 in comparison with scramble control transfection

To validate the potential interaction between miR-125b and STAT3, we constructed luciferase reporter plasmids encompassing the WT or MUT 3′-UTR of STAT3 (Fig. 2a upper panel). The dual-luciferase reporter assay verified that the luciferase activity of the STAT3-WT-3′-UTR plasmid in the CSCC cell line SCC13 was reduced significantly by upregulation of miR-125b, while the luciferase activity of STAT3-MUT-3′-UTR did not change (Fig. 2a lower panel).

We also observed the influence of miR-125b upregulation on the expression levels of STAT3 in CSCC cells. Although miR-125 mimics did not influence the mRNA levels of STAT3 (data not shown), STAT3 protein expression decreased remarkably in A431, SCC13 and SCL-1 cells after transfection with miR-125b mimics, compared with the scrambled control (Fig. 2b).

We also verified the direct interaction between miR-125b and STAT3 mRNA via immunoprecipitation and quantitative PCR. Anti-human Ago2 antibody was used for RNA immunoprecipitation and quantitative RT-PCR was used to test the relative expression of STAT3 mRNAs from Ago2-IP fractions. STAT3 mRNA relative expression levels increased significantly in cells transfected with miR-125b mimics and STAT3-WT plasmids (Fig. 2c). These findings indicate that miR-125b may directly target STAT3 in CSCC cells.

### The effect of miR-125b overexpression or STAT3 downregulation on CSCC cells

Based on these results, we supposed that STAT3 is a direct target of miR-125b. Therefore, we constructed STAT3 shRNA and control vectors to observe the effect of the miR-125b mimic and STAT shRNA on CSCC cell proliferation ability, cycle progression and apoptosis.

**Fig. 3:**
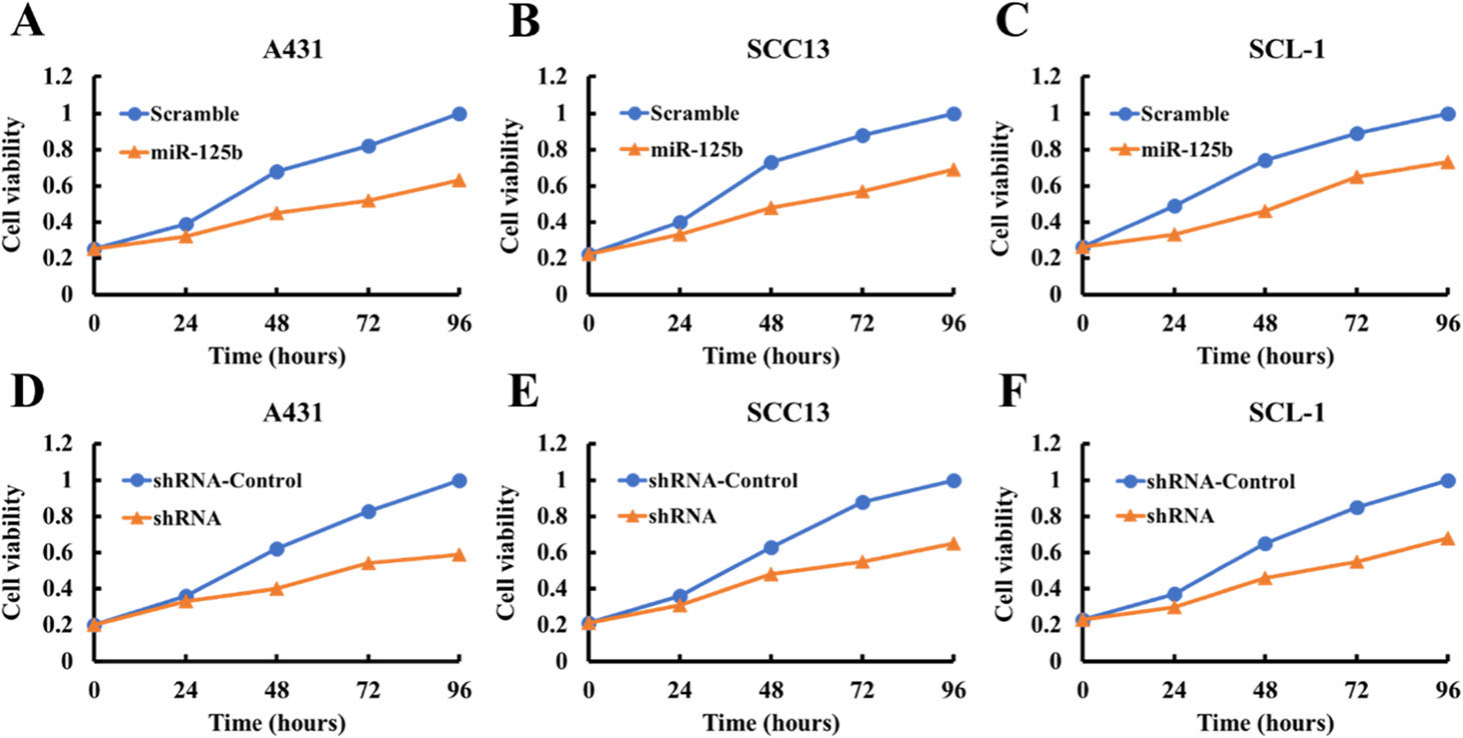
The knockdown of STAT3 inhibits CSCC cell proliferation. This was assessed using the MTT assay. **a**, **b** and **c** STAT3 knockdown by miR-125b mimics inhibits A431, SCC13 and SCL-1 cell proliferation. **d**, **e** and **f** STAT3 knockdown by shRNA inhibits A431, SCC13 and SCL-1 cell proliferation. Data are the means ± SD of three independent experiments

MTT assay results showed that the ectopic expression of miR-125b after miR-125b mimic transfection attenuated the proliferation ability of CSCC cells (A431, SCC13 and SCL-1; Fig. 3a through c). Similarly, the shRNA-mediated downregulation of STAT3 also significantly suppressed cell proliferation capacity in A431, SCC13 and SCL-1 cells (Fig. 3d through f). The results of simple cell count assays were similar to those of the MTT assay (data not shown).

**Fig. 4:**
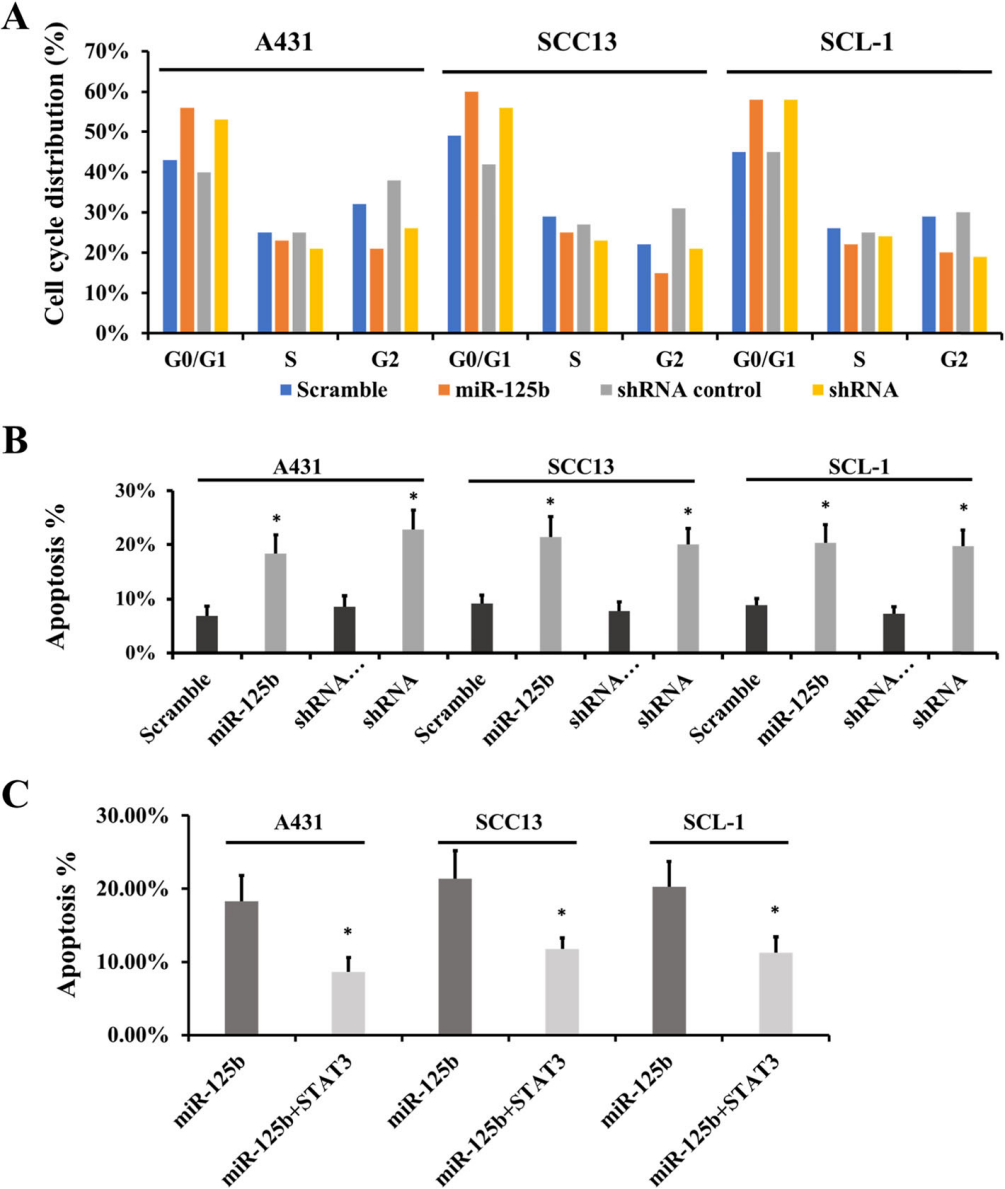
Knockdown of STAT3 inhibits CSCC cell cycle progression or promotes cell apoptosis. **a** STAT3 knockdown by the miR-125b mimic and shRNA inhibits A431, SCC13 and SCL-1 cell cycle progression. **b** STAT3 knockdown by the miR-125b mimic or shRNA promotes A431, SCC13 and SCL-1 cell apoptosis. **c** Additional STAT3 supplementation can attenuate the apoptosis induced by the miR-125b mimic. Data are expressed as means ± SD of three independent experiments. *p < 0.05, compared with the miRNA scramble or shRNA control

Flow cytometry results also confirmed that both miR-125b overexpression and STAT3 downregulation could inhibit the growth of CSCC cells (A431, SCC13 and SCL-1; Fig. 4a). An increase of cell numbers in the G0/G1 phase was observed when CSCC cells were transfected with miR-125b mimics or STAT3 shRNA. This was accompanied by a decrease of cell numbers in the S and G2 phases. This impact on the cell cycle pattern suggests that miR-125b and STAT3 play regulatory roles in cell cycle progression, and thus influence CSCC cell proliferation.

Also using flow cytometry, we observed the influence of the miR-125b–STAT3 axis on CSCC cell apoptosis. STAT3 downregulation by the miR-125b mimic or STAT3 shRNA increased the number of CSCC apoptotic cells compared with the impact of the scramble or shRNA control (Fig. 4b), indicating that miR-125b and STAT3 are involved in CSCC cell apoptosis. Moreover, additional STAT3 supplementation can attenuate the apoptosis induced by the miR-125b mimic (Fig. 4c).

These results further confirm STAT3 as a direct target of miR-125b. Therefore, miR-125b at least partially inhibits CSCC cell proliferation and promotes CSCC cell apoptosis by targeting STAT3.

### Downstream genes of the miR-125b–STAT3 axis in CSCC cells

**Fig. 5:**
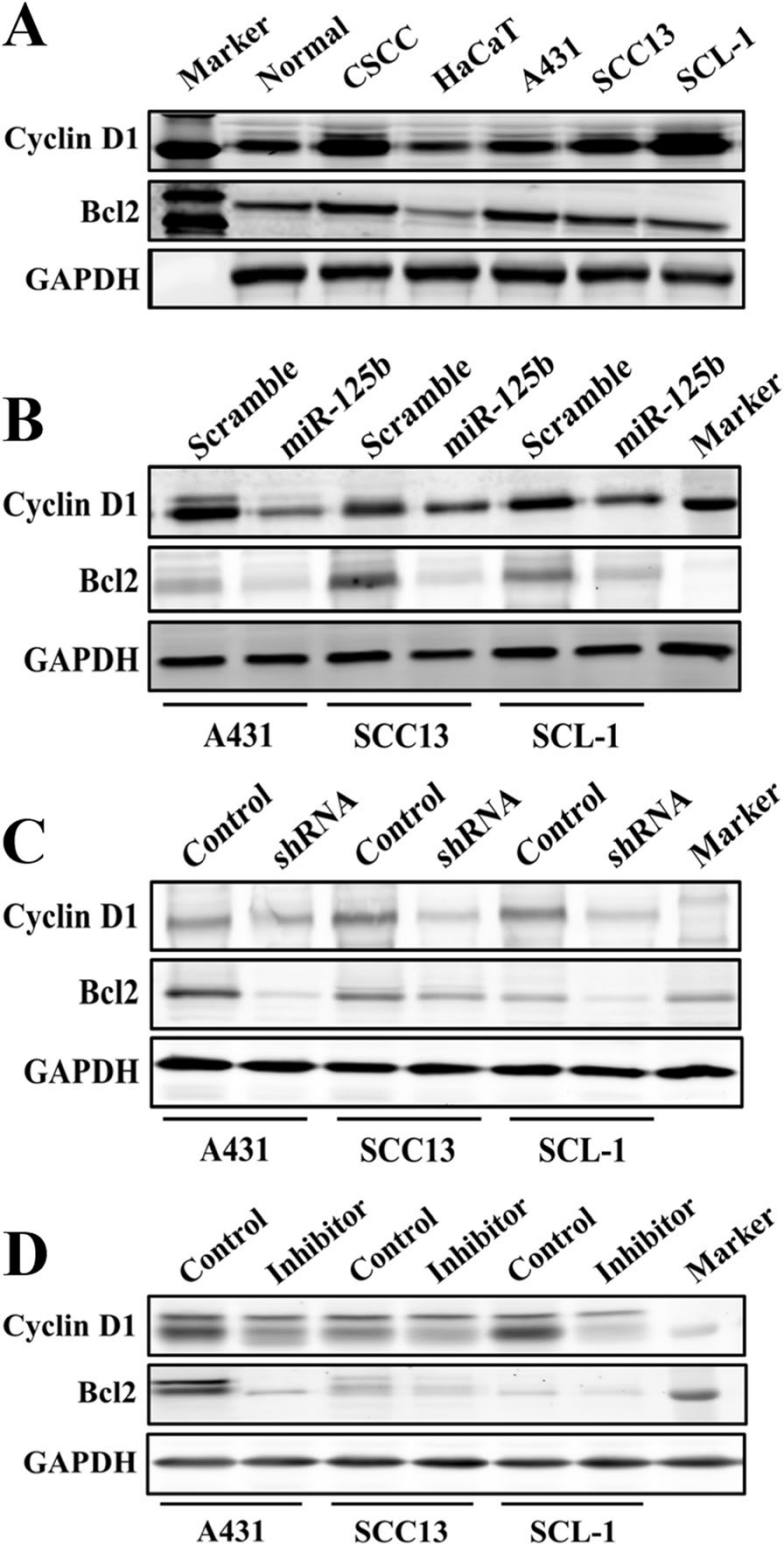
Cyclin D1 and Bcl2 are the downstream targets of miR-125b/STAT3. **a** The expressions of cyclin D1 and Bcl2 in CSCC and normal tissues and cells. **b**, **c** and **d** The miR-125 mimic, STAT3 shRNA and inhibitor could inhibit the expression of cyclin D1 and Bcl2 in CSCC cells

We also explored the target genes of the miR-125b–STAT3 axis in CSCC cells. Western blotting results showed that cyclin D1 and Bcl2 protein levels increased to varying degrees in CSCC tissues and cell lines compared with normal tissues and cells (Fig. 5a). The miR-125b mimic, STAT3 shRNA or STAT3 inhibitor HO-3867 could deteriorate the overexpression of cyclin D1 and Bcl2 (Fig. 5b through d). These findings indicate that cyclin D1 and Bcl2 may be the downstream genes of the miR-125b–STAT3 axis in CSCC cells. The miR-125b–STAT3 axis regulates CSCC cell proliferation and apoptosis, probably through targeting cyclin D1 and Bcl2.

## Discussion

In recent decades, dysregulation of miRNA expression has been demonstrated to occur during the development and progression of CSCC and other types of human cancer [17–21]. For instance, inhibition of miR-21 could suppress CSCC cell growth and invasion by targeting two tumor suppressors, PDCD4 and PTEN [22]. MicroRNA-31 is overexpressed in CSCC and regulates cell motility and colony formation ability [23]. Downregulation of miR-34a is related to the aggressive progression of CSCC [24].

MiR-125b has been reported to act as a tumor repressor or promoter [25]. For example, miR-125b shows reduced expression in ovarian cancer, but is overexpressed in colorectal tumors [26, 27]. Xu et al. reported that miR-125b has lower expression in CSCC cells than in healthy skin cells, and miR-125b overexpression could suppress CSCC cell proliferation, migration and invasion by targeting matrix metallopeptidases 13 and 7 and mitogen-activated protein kinase 7 [13]. These findings suggest that miR-125b plays a tumor-suppressor role in CSCC. However, its role in CSCC is not completely clear.

In our study, a significant downregulation of miR-125b was identified in CSCC tissues and cell lines. Moreover, miR-125b mimics inhibited the colony formation, migration and invasion ability of SCC13 cells (a CSCC cell line). This suggests that miR-125b negatively regulates the growth of CSCC. These results are consistent with those of previous studies, showing that miR-125b plays the role of tumor suppressor in CSCC.

STAT3 belongs to the STAT family, which is closely associated with abnormal cell proliferation, apoptosis, tumorigenesis, invasion and metastasis [28–31]. The miR-125b–STAT3 axis has a reported involvement in tumor cell proliferation, apoptosis and migration, for example, in osteosarcoma cells [17], liver, lung and colorectal cancer cells [32], melanoma cells [33], laryngeal and oral squamous cell carcinoma cells [34, 35], and cervical cancer cells [36].

However, the role of the miR-125b–STAT3 axis in CSCC remains unclear. Here, we observed that miR-125b downregulation in CSCC tissues and cell lines is inversely associated with STAT3 protein levels but not mRNA levels. Furthermore, the luciferase reporter gene assay and Ago2 immunoprecipitation–quantitative PCR identified that miR-125b inhibited STAT3 expression by directly targeting the 3′-UTR in CSCC cells. These findings verify that the miR-125b–STAT3 axis is involved in CSCC and that miR-125b inhibits STAT3 expression by inhibiting translation rather than by mRNA degradation.

To explore the exact functional roles of the miR-125b–STAT3 axis in CSCC cells, we performed MTT and flow cytometry assays. The results revealed that STAT3 knockdown by miR-125b mimic or by shRNA inhibits cell proliferation and cell cycle progression and promotes apoptosis. Our study suggests that the miR-125b–STAT3 axis regulates cell proliferation, cell cycle progression and apoptosis of CSCC cells.

Many downstream genes of STAT3 have been identified in human cancers, including genes associated with cell cycle (cyclin D1 and cMyc) [37, 38], apoptosis (Bcl2-xL and Mcl-1) [39, 40] and metastasis (MMP1 and MMP2) [16, 41]. Consistent with previous studies, our findings verify that cyclin D1 and Bcl2 are downstream targets of the miR-125b–STAT3 axis.

Although recent studies have reported that miR-125b and STAT3 are independently involved in CSCC development and progression, we have here confirmed the association between miR-125b and STAT3 in CSCC cells. Further studies should be performed to identify the potential role of the miR-125b–STAT3 axis in CSCC diagnosis and treatment.

## Conclusions

Our studies identify miR-125b as a tumor suppressor in CSCC tumorigenesis and progression. It targets the STAT3 pathway to regulate cell proliferation, cell cycle progression and apoptosis. This observation adds to the understanding about the molecular mechanisms underlying the development and progression of CSCC. Therapies aimed at activating miR-125b or inhibiting STAT3 pathway signaling deserve exploration as potential treatments for CSCC.

## Data Availability

The data supporting the conclusions of this article are available from the corresponding author on reasonable request.

## Abbreviations

CSCC: Cutaneous squamous cell carcinoma
miR-125b: MicroRNA-125b
miRNAs: MicroRNAs
MUT: Mutant type
SD: Standard deviation
STAT3: Signal transducer and activator of transcription 3
UTR: Untranslated region
WT: Wild type
